# Structure internal of the dimension Human Person of the Questionnaire of Health Vulnerability in Heart Failure

**DOI:** 10.1590/1980-220X-REEUSP-2022-0117en

**Published:** 2022-12-16

**Authors:** Virna Ribeiro Feitosa Cestari, José Wicto Pereira Borges, Raquel Sampaio Florêncio, Thiago Santos Garces, Vera Lúcia Mendes de Paula Pessoa, Thereza Maria Magalhães Moreira

**Affiliations:** 1 Universidade Estadual do Ceará, Programa de Pós-Graduação em Cuidados Clínicos em Enfermagem e Saúde, Fortaleza, CE, Brazil.; 2 Universidade Federal do Piauí, Programa de Pós-Graduação em Enfermagem, Teresina, PI, Brazil.

**Keywords:** Heart Failur, Health Vulnerabilit, Validation Stud, Psychometric, Factor Analysis, Insuficiencia Cardíaca, Vulnerabilidad en Salud, Estudio de Validación, Psicometría, Análisis Factorial, Insuficiência Cardíaca, Vulnerabilidade em Saúde, Estudo de Validação, Psicometria, Análise Fatorial

## Abstract

**Objective:**

to analyze the evidence of validity of the internal structure of the dimension Human Person of the Questionnaire of Health Vulnerability in Heart Failure.

**Method:**

psychometric study with 1,008 people with heart failure, in a tertiary healthcare institution located in Northeastern Brazil. The internal structure was assessed by exploratory factor analysis with a polychoric correlation matrix, followed by confirmatory factor analysis to verify the quality of the model fit. Internal consistency was measured by composite reliability (CR) and McDonald’s omega (ω).

**Results:**

psychometric parameters revealed 22-item model, distributed in five factors, total explained variance of 64.9%, factor loadings (0.38 to 0.97) and adequate communalities (0.20 ≤ h2 ≤ 0.98) and acceptable indicators of precision (0.79 ≤ ORION ≤ 0.98), representativeness (0.89 ≤ FDI ≤ 0.99), sensitivity (1.92 ≤ SR ≤ 7.07), factor expectancy (88.3% ≤ EPTD ≤ 97.9%), replicability (0.82 ≤ H-latent ≤ 0.97; 0.81 ≤ H-observed ≤ 0.87) and reliability (CR = 0.92 and ω = 0.83). Adequate adjustment quality was achieved (TLI = 0.99; CFI = 0.99; GFI = 0.99; RMSEA = 0.04 and RMSR = 0.04).

**Conclusion:**

We obtained an instrument with good evidence of internal structure validity for construct measurement.

## RESUMO

**Objetivo:**

analisar as evidências de validade de estrutura interna da dimensão Pessoa humana do Questionário de Vulnerabilidade em Saúde na Insuficiência Cardíaca.

**Método:**

estudo psicométrico com 1.008 pessoas com insuficiência cardíaca, em instituição de saúde terciária situada no Nordeste brasileiro. A estrutura interna foi avaliada por meio de análise fatorial exploratória com matriz de correlação policórica, seguida da confirmatória, para atestar a qualidade do ajuste do modelo. A consistência interna foi medida pela fidedignidade composta (FC) e ômega de McDonald (ω).

**Resultados:**

os parâmetros psicométricos revelaram modelo de 22 itens, distribuídos em cinco fatores, variância total explicada de 64,9%, cargas fatoriais (0,38 a 0,97) e comunalidades (0,20 ≤ h^2^ ≤ 0,98) adequadas, e indicadores aceitáveis de precisão (0,79 ≤ ORION ≤ 0,98), representatividade (0,89 ≤ FDI ≤ 0,99), sensibilidade (1,92 ≤ SR ≤ 7,07), espera do fator (88,3% ≤ EPTD ≤ 97,9%), replicabilidade (0,82 ≤ H-latente ≤ 0,97; 0,81 ≤ H-observado ≤ 0,87) e confiabilidade (FC = 0,92 e ω = 0,83). Alcançou-se qualidade de ajustamento adequada (TLI = 0,99; CFI = 0,99; GFI = 0,99; RMSEA = 0,04 e RMSR = 0,04).

**Conclusão:**

obteve-se um instrumento com boas evidências de validade de estrutura interna para mensuração do constructo.

## RESUMEN

**Objetivo:**

analizar las evidencias de validez de la estructura interna de la dimensión Persona Humana del Cuestionario de Vulnerabilidad Sanitaria en la Insuficiencia Cardíaca.

**Material y método:**

estudio psicométrico con 1.008 personas con insuficiencia cardíaca, en una institución de salud terciaria ubicada en el Nordeste de Brasil. La estructura interna fue evaluada por análisis factorial exploratorio con matriz de correlación policórica, seguido de análisis factorial confirmatorio para verificar la calidad del ajuste del modelo. La consistencia interna se midió mediante la fiabilidad compuesta (FC) y el omega de McDonald (ω).

**Resultados:**

los parámetros psicométricos revelaron un modelo de 22 ítems, distribuidos en cinco factores, una varianza total explicada del 64,9%, cargas factoriales (0,38 a 0,97) y comunalidades adecuadas (0,20 ≤ h2 ≤ 0,98) e indicadores de precisión aceptables (0. 79 ≤ ORION ≤ 0,98), representatividad (0,89 ≤ FDI ≤ 0,99), sensibilidad (1,92 ≤ SR ≤ 7,07), expectativa de factor (88,3% ≤ EPTD ≤ 97,9%), replicabilidad (0,82 ≤ H-latente ≤ 0,97; 0,81 ≤ H-observado ≤ 0,87) y fiabilidad (CR = 0,92 y ω = 0,83). Se alcanzó una calidad de ajuste adecuada (TLI = 0,99; CFI = 0,99; GFI = 0,99; RMSEA = 0,04 y RMSR = 0,04).

**Conclusión:**

Se obtuvo un instrumento con buena evidencia de validez de estructura interna para la medición del constructo.

## INTRODUCTION

Heart failure (HF) is a syndrome presenting high morbidity and mortality worldwide and with a high incapacitating potential^(1)^, which is expressed in different contexts. The burden of HF reflects the numerous conditions of health vulnerability (HV) experienced by the person who, to coexist with the disease, is immersed in a series of rules that dictate daily self-care actions for control, monitoring and management of symptoms.

To help and support disease management, patients, their families/caregivers and health professionals use instruments that propose the measurement of several constructs^(2)^ that intervene in the HV. To study a health issue from the HV perspective is to seek new ways to better understand a health problem, adopting and prioritizing the holistic approach and integral understanding of phenomena, as opposed to the isolated analytical process.

In the context of HF, HV is a phenomenon intrinsic to human existence and involves interdependent dimensions: Human Person, co-presences and Care^(3)^. It is in the dimension of the human person that the subject understands her/his health condition in order to react to situations that place him in vulnerability^(4)^.

Despite the relevance of understanding the HV in this population, a proper instrument to measure it has not yet been identified. With the intention of filling this gap, two studies are highlighted: the first one identified the constituent elements of the HV of the person with HF^(5)^ and the second one focused on the construction and validation of a bank of items^(6)^. As a result, 49 theoretical elements guided the elaboration of 110 items, divided into three dimensions and validated by 19 health professionals specialized in HF, with content validity index (CVI) per item ≥0.78, without significant disagreement between experts (p > 0.05) as to the score, attested by the binomial exact test, and total CVI 0.99^(6)^.

In the items bank developed, the Human Person dimension comprised 66 items related to the subject, organized in the subdimensions: socioeconomic, demographic and clinical profile; learning; mental health and health behaviors^(6)^. In this research, the evidence of validity of the internal structure of the human person dimension of the questionnaire was analyzed. Nevertheless, even though the evidence of validity related to the content by experts has been satisfactory, it is also necessary to observe the evidence of validity of the internal structure of the instrument, in order to assess the agreement between items and construct^(7)^, and thus obtain a valid and reproducible parameter.

To search for evidence of validity of an instrument that allows measuring aspects related to the dimension of the Human Person of the subject with HF is an opportunity to conceive its plurality and plan the integral and continuous care, in addition to driving policies to improve care, in a much more effective direct contribution to health. Thus, this study aimed to analyze the evidence of validity of the internal structure of the dimension Human Person of the Questionnaire of Health Vulnerability in Heart Failure (QVSIC-Human Person).

## METHODS

### Study Design

Psychometric study, focused on the analysis of evidence of internal structure^(7)^ of the QVSIC-Human Person.

### Setting

The study was developed with patients followed-up in a tertiary healthcare institution of reference in the care of people with cardiopulmonary diseases, located in the city of Fortaleza, Ceará, Brazil.

### Sample Definition

The target population of the study consisted of 1,008 people with a medical diagnosis of HF, selected by convenience. HF patients aged ≥ 18 years, followed or admitted to the wards, outpatient clinics, cardiac rehabilitation and emergency of the institution were included, and those who did not communicate verbally were excluded.

In the planning of this research, the instrument was fully applied, considering the 110 items. However, for the present study, validity evidence was analyzed only for the human dimension of the instrument, which contains 66 items.

Considering the extension of the general item bank and with the intention of preserving heterogeneity and getting respondents that covered the whole construct, 1,100 people were invited. However, 92 participants were lost due to incomplete responses to the items. The 1,008 participants guaranteed an average of 9.2 observations for each item of the general instrument; when related to the 66 items of the Human Person dimension, the average of participants was 15.3 per item. The sample size of psychometric studies is estimated based on the number of items, which show ratios of 10:1 or more^(8)^. Therefore, the number of participants was adequate based on literature recommendations.

### Data Collection

Data were collected in the period from June 2019 to January 2020, through individual interviews conducted at the healthcare facility. Patients were approached in the transplant and HF unit (UTIC in the Portuguese acronym) and inpatient and cardiac rehabilitation and emergency sectors of the institution. Patients were picked up and the objectives and relevance of the research were explained. Those who agreed to participate in the study signed the Informed Consent Form, provided in two copies. The patients were then referred to a reserved room for privacy assurance.

The research coordinator and a previously trained nursing student, scientific initiation scholarship holder, participated in the collection. Two instruments were used: a questionnaire of sociodemographic data (gender, age, region of residence, color, education, family income, number of people in the same household, whether they have a paid activity and whether they receive benefits related to the disease), clinical (HF etiology, functional class and number of hospitalizations in the last year) and behavioral (smoking, alcohol consumption and whether they engage in physical activity); and a bank consisting of 66 items relating to the peculiarities of the person with HF in a vulnerable situation. Among the 66 items, 14 have a dichotomous response pattern (yes/no) and 52 are polytomous, using a Likert-type scale of five points ranging from 1 to 5 (from never to always).

### Data Analysis and Treatment

Data were organized in Microsoft Office Excel® spreadsheets and exported to the statistical programs Factor (version 11.05.01) and R (version 3.6.2). To characterize the sociodemographic, clinical, and behavioral data of the research participants, measures of central tendency and dispersion of quantitative variables and simple frequency and percentage of qualitative variables were calculated. Normality was assessed using the Kolmogorov-Smirnov test.

Exploratory Factor Analysis (EFA) was used to validate the internal structure of the QVSIC-Human Person. The suitability of the data for factor analysis was examined using the Kaiser-Meyer-Olkin value (KMO > 0.60) and Bartlett’s test of sphericity. High KMO values indicate the adequacy of the EFA. If the calculated error probability (p-value) of the sphericity test is below 0.05, it is concluded that the correlation or covariance matrix is not a unitary matrix, so factor analysis can be applied^(9)^.

The dimensionality of the instrument was tested by applying Parallel Analysis using the Parallel Analysis Optimal Implementation technique^(10)^. The robustness of the test was determined from the bootstrap association, with extrapolation of the sample to 1000 cases. This data resampling procedure is performed in order to obtain confidence intervals of the evaluated parameters^(11)^.

The factors were extracted by the Robust Unweighted Least Squares (ULS) method with polychoric correlation, suitable for polytomous data, and matrix residual reduction^(12)^, and Robust Promin rotation^(13)^. To complement the testing of the number of factors of the total instrument, unidimensionality/multidimensionality techniques were applied: Unidimensional Congruence (UniCo > 0.95), Explained Common Variance (ECV > 0.85) and Mean of Item Residual Absolute Loadings (MIREAL < 0.30)^(14)^.

The division of the bank to ensure representativeness was performed by the Solomon method, which optimally divides the sample into two equivalent halves to ensure that all possible sources of variation are included in the two subsamples, as attested by the Ratio Communality Index (RCI): the closer to 1.0, the more equivalent are the subsamples and, consequently, the more representative^(15)^.

Maintenance or removal of items from the model was done considering: Polycoric Matrix convergence; percentage of covariance destroyed for each item (PCDi); correlation above 0.2 with two other items (those below were eliminated); kurtosis and asymmetry; communalities (h2 > 0.40) and factor load values – those >0.30 were maintained and *heywood cases* (negative variance estimates or factor load estimates equal to or above 1.00) and with double saturation were excluded. Communality is an important metric for indicating how much each variable is explained by all the factors. Factorial loadings, on the other hand, indicate how much each factor explains each variable^(8)^. It is reiterated that the estimated factorial solutions were evaluated based on the theoretical reasonableness and interpretation of the factors according to the theoretical assumptions of the HV in the context of HF.

Regarding the quality and effectiveness of factor score estimates, accuracy (Overall Reliability of fully Informative prior Oblique N-EAP scores – ORION > 0.70), representativeness of the latent trait and effectiveness of factor estimation (Factor Determinacy Index – FDI > 0.80^(16)^, sensitivity (Sensitivity Ratio – SR > 2.0), expected percentage of the factor (Expected Percentage of true Differences – EPTD > 90%) and replicability (Generalized G-H Index > 0.80)^(14)^ were assessed.

To test the adjustment of the data to the five-factor structure, confirmatory factor analysis (CFA) was performed. The following indices were used to evaluate the quality of adjustment of the model and their respective 95% confidence intervals: Tucker Lewis Index (TLI > 0.90); Comparative Fit Index (CFI > 0.94); Goodness of Fit Index (GFI > 0.95); Adjusted Goodness of Fit Index (AGFI > 0.93); Root Mean Square Error of Approximation (RMSEA < 0.07); and Root Mean Square of Residuals (RMSR < 0.08)^(17)^.

Factor reliability was checked by McDonald’s Omega coefficient (ω), to verify the maintenance of the Tau equivalence principle; and composite reliability (CR), calculated by the Composite Reliability Calculator, based on standardized factor loadings and error variances (www.thestatisticalmind.com), whose reference values adopted for these measures were <0.6 low; between 0.6 and 0.7, moderate; and between 0.7 and 0.9 high reliability^(8,18–19)^. We chose to include the WC to increase the reliability of interpretation, because numerous inconsistencies of reliability using Cronbach’s alpha have been reported^(20)^.

### Ethical Aspects

The study received clearance from the Research Ethics Committee in the year 2019, with opinion no. 3,563,547, in line with what is recommended by Resolution 466/2012, of the National Health Council, and from those responsible for the institution. All participants signed the Informed Consent Form (ICF).

## RESULTS

The items were answered by 1. 008 people with HF, mostly males (634; 62.9%), mixed race (862; 85.5%), with a minimum age of 19 and maximum of 93 years (56 ± 13.9 years), from the Northeast region (993; 98.5%), with varied level of education – predominance of subjects with incomplete elementary school (309; 30.7%), and from social class D (R$ 1,874.01 to 3,748.00) (615; 61%). Most lived with one person (602; 59.7%); 820 (81.3%) still had a paid job, 730 (72.4%) were retired, and 710 (70.4%) received government benefits because of the disease.

Initially, eight items were excluded because they had zero variance. In model 1, the results of the analysis of the 58 items revealed a negative matrix, with a bad percentage of destruction of each item (45.4%) and unacceptable KMO, which made further analysis impossible. In model 2, after exclusion of the 14 dichotomous items, we obtained a positive matrix, mediocre KMO (<0.60), and unsatisfactory quality of fit. The results supported the removal of 22 items for presenting inadequate communalities, factor weights below what is considered adequate and double saturation. With the elimination of these items, we proceeded with a new analysis (model 3), with 22 items and adequate adjustment of the data, being considered the best and most parsimonious model tested.

Model 3 presented good sample adequacy, attested by Bartlett’s tests of sphericity (11499.5; gl = 231 and p < 0.001), KMO (0.65 [95%CI = 0.54–0.85]) and RCI (0.98), which suggested interpretability of the correlation matrix of the items and representativeness of the subsamples. Parallel analysis indicated five representative factors of the items, with their respective explained variances: factor 1 – 19.1%, factor 2 – 14.4%, factor 3 – 12.6%, factor 4 – 10.9%, and factor 5 – 7.9%. The multidimensionality of the instrument was confirmed by the UniCo (0.57 [95%CI = 0.44–0.74]), ECV (0.57 [95%CI = 0.55–0.60]) and MIREAL (0.27 [95%CI = 0.19–0.31]) indices.

Table 1 details the factors and descriptive results of the QVSIC-Human Person. The first factor (06 items – 30, 31, 32, 33, 35, and 36), encompassed aspects related to fluid control and diet, qualified as fluid and food intake. The second factor (04 items – 22, 23, 25, and 26) involved items related to sleep difficulty and its consequences and daily activities, designated as Sleep and daily activities. The third (03 items – 56, 57, and 58) was composed of items regarding psychoemotional issues, termed Mental health. The fourth (05 items – 05, 08, 11, 12, and 13), with items related to education and knowledge about the disease and treatment, named Functional health literacy. And finally, the fifth factor (04 items – 14, 15, 16, and 17) comprised items related to difficulty breathing, concentration, memory problems, and fatigue, named Signs and symptoms (Table 2).

**Table 1.** Factorial loads, communalities, shortness and reliability of the final model with five factors – Fortaleza, CE, Brazil, 2021.

Items	Factorial Loads					h^2^	K	CR
	F1	F2	F3	F4	F5			
05				0.60		0.40	0.02	0.75
08				0.45		0.26	0.32	
11				0.76		0.57	0.86	
12				0.71		0.52	0.61	
13				0.54		0.31	−0.56	
14					0.74	0.55	4.94	0.86
15					0.78	0.61	1.64	
16					0.81	0.66	−0.42	
17					0.80	0.65	−0.62	
22		0.95				0.92	−1.75	0.97
23		0.97				0.95	−1.57	
25		0.94				0.88	−0.94	
26		0.92				0.84	−0.76	
30	0.38					0.20	−0.66	0.76
31	0.63					0.40	−0.01	
32	0.60					0.35	−0.54	
33	0.71					0.50	−0.69	
35	0.66					0.43	−1.18	
36	0.55					0.32	−0.86	
56			0.79			0.64	−0.33	0.86
57			0.80			0.64	−0.62	
58			0.88			0.98	1.10	
% Variance	19.1	14.4	12.6	10.9	7.9	Total variance 64.9%		

F1: Water and food intake; F2: Sleep and daily activities; F3: Mental health; F4: Functional health literacy; F5: Signs and symptoms; h2: communality; K: kurtosis; α: Cronbach’s alpha; CR: composite reliability.

**Table 2.** Model fit indices.

Estimates	Model 1	Model 2	Model 3	CI95%*
Polycoric Matrix	Negative	Positive	Positive	−
*Kaiser–Meyer–Olkin*	0.00	0.58	0.65	−
*Tucker Lewis Index*	−	0.82	0.99	0.95–0.99
*Comparative Fit Index*	−	0.90	0.99	0.98–0.99
*Goodness of Fit Index*	−	0.89	0.99	0.95–0.99
*Adjusted Goodness of Fit Index*	−	0.91	0.98	0.89–0.99
*Root Mean Square Error of Approximation*	−	0.08	0.04	0.04–0.06
*Root Mean Square of Residuals*	−	0.08	0.04	0.04–0.07

*CI95%: Confidence Interval 95% for Model 3.

It was considered that the values of the communalities of the items represented the variability explained by the factors and the kurtosis did not indicate severe deviations from the normal distribution of responses and, consequently, psychometric sensitivity. Also, the factors presented adequate values of reliability, and a reliability of 0.92, which ratified their internal consistency. The model presented ω = 0.83.

Table 2 shows the fit indices of the tested models and reveals the quality of model 3 over the others, expressed by the values found in the exploratory and confirmatory factor analyses.

Table 3 specifies the quality and effectiveness of the factor score estimates of the QVSIC-Human Person. The questionnaire proved adequate with regard to accuracy (0.79 ≤ ORION ≤ 0.98), representativeness (0.89 ≤ FDI ≤ 0.99), sensitivity (1.92 ≤ SR ≤ 7.07), factor expectancy (88.3% ≤ EPTD ≤ 97.9%), and replicability (0.82 ≤ H-latent ≤ 0.97; 0.81 ≤ H-observed ≤ 0.87). It should be remarked that although the factors Education and functional health literacy and Water and food intake present SR and EPTD below the reference value, the model shows several indicators that attest to its quality.

**Table 3.** Quality and effectiveness of factor score estimates – Fortaleza, CE, Brazil, 2021.

Factors	ORION	FDI	SR	EPTD	G-H *Index*	
					H-latent	H-observed
Education and functional health literacy	0.79	0.89	1.92	88.3%	0.94 (0.87–0.95)	0.87 (0.85–0.80)
Signs and symptoms	0.98	0.99	6.69	97.7%	0.97 (0.84–0.93)	0.86 (0.84–0.87)
Sleep and daily activities	0.98	0.99	7.07	97.9%	0.96 (0.93–0.97)	0.84 (0.82–0.85)
Water and Food Intake	0.79	0.89	1.94	88.4%	0.82 (0.80–0.84)	0.81 (0.78–0.82)
Mental Health	0.87	0.93	2.57	91.1%	0.92 (0.91–0.94)	0.82 (0.81–0.85)

ORION: *Overall Realibity of fully-Informative prior Oblique N-EAP scores* (>0,70); FDI: *Factor Determinacy Index* (>0,80); Sensitivity Ratio (SR > 2,0); Expected Percentage of True Differences (EPTD > 90%).

**Chart 1.**
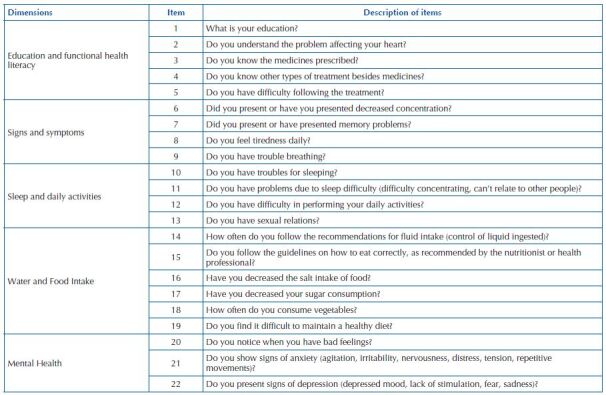
Questionnaire of Health Vulnerability in Heart Failure, human dimension – Fortaleza, CE, Brazil, 2021.

In view of the results obtained, Chart 1 shows the dimensions and corresponding items of the QVSIC-Human Being.

The evaluated indicators pointed to a five-dimensional model with evidence of consistent internal structure for measuring the construct human vulnerability in the context of HF.

## DISCUSSION

Validity based on internal structure represents the degree to which the structure of correlations among items corresponds to the structure of the construct that the test proposes to measure. Therefore, assessing evidence of the internal structure of measurement instruments is a complex procedure, which requires a series of requirements, such as the factorial structure of the instrument and reliability, which are crucial for it to be effective^(7,21)^.

In this study, the sample proved to be adequate and representative for further factor analysis, as evidenced by Bartlett’s tests of sphericity, KMO, and RCI. The EFA is performed to explore the underlying dimensionality of a set of variables^(8,12)^ and can be performed in several statistical softwares. However, Factor stands out by enabling the use of parallel analysis and other dimensionality indicators. The advantage of parallel analysis lies in the fact that the technique is based on samples and not on the population; it allows the random construction of a hypothetical set of correlation matrices of variables, using the same dimensionality of the real data set as a basis, and consequently it decreases the probability of erroneous retention of items, as it considers sampling error and minimizes the influence of sample size and factorial loads of the items^(22)^.

To obtain a valid model, it was necessary to review the instrument regarding the irregularity of the items (dichotomous and polytomous). Problems with factor analysis of dichotomous variables have already been recognized in the literature. Although dichotomous items can be submitted to factor analysis using standard techniques, the results may be biased, such as overestimated values of explained variance and instrument quality^(23)^. In this sense, alternative approaches have been suggested for factor analysis of dichotomous data.

Factor analysis showed that the HV construct in the human person with HF is organized into the factors Education and functional health literacy, Signs and symptoms, Sleep and daily activities, Water and food intake and Mental health. The five factors evidenced by the factor analysis correlation matrix were congruent with the concept of HV in the person with HF and its constituent elements^(6)^: in the dimension of the Human Person, the HV is multifactorial and incorporates social, educational, physiological, clinical, psychological and behavioral attributes. These findings demonstrate the importance of breaking with the merely technical care, focused only on the pathological process, and to adopt preventive and health-promoting measures that allow the understanding of the multiple facets of the subject in his vulnerable condition.

The link between the theoretical and empirical structure of the instrument is confirmed by reference authors^(24)^ who highlight the subject as one of the central nuclei in the interpretation of the HV, for understanding that this is a product of power relations, and can obtain means for awareness and resist the HV through agencying. This way, there is the possibility of movement towards a better condition of health and life, that is, less vulnerability.

The multidimensionality of the model demonstrates the various peculiarities of the HV of the person with HF, so a deep analysis of the factors is necessary. It is essential for professionals to have means to help identify and assess situations of vulnerability and thus contribute to the direction and planning of care. Therefore, the instrument developed stands out for allowing the professional to obtain from the subjects their perceptions about their health conditions by containing items that address the understanding of the disease and how it impacts their quality of life.

The indicators that were anchored in the first factor are related to eating habits and water intake, with items related to the control of fluid intake, correct diet, decreased consumption of salt and sugar, and difficulties in maintaining a healthy diet. Sodium restriction, supplementation of dietary fats, fatty acids, proteins, amino acids and vitamin and mineral micronutrients, as well as strategies such as nitrates in beet juice have shown promising results in the outcomes of HF, being therefore pointed out as sustainable therapies^(25)^. In this sense, the items enabled the developing of eating habits that can influence a higher or lower level of vulnerability.

The second factor supported items on sleep and daily activities, including sexual intercourse. Common HF symptoms such as dyspnea, fatigue, low exercise tolerance and fluid retention strongly affect sleep quality, contributing to chronic insomnia^(26)^. However, an experimental study revealed that sexual interactions improve diastolic function, reduce HF-associated edema, alter the transcription of heart contractile protein genes, and decrease plasma levels of testosterone, which leads to prolonged survival^(27)^.

The third factor brought items related to mental health, which identifies the subjects’ perception about their feelings and signs of anxiety or depression, frequent conditions that exacerbate vulnerability by interfering in the physical and psychological state, associated with poor quality of life^(28)^. The fourth factor covered items on education, subject’s understanding of the disease, and knowledge of the therapeutics. Knowledge and active participation of the patient in his treatment is essential to optimize self- management^(29)^ and, consequently, lower levels of vulnerability.

The fifth and last factor involves items on the presentation of signs and symptoms of HF, such as decreased concentration, memory problems and physical signs, the main ones being fatigue and dyspnea. The gradual or sudden worsening of the disease symptoms causes frequent decompensations that lead to progressive deterioration of cardiac function^(30)^, impacting negatively on the subject’s life contexts.

All factors presented items with satisfactory factor loadings (there was no violation of the limits) and saturating in a single factor as well as kurtosis considered statistically acceptable. Factor loadings indicate how much a factor explains a variable, while communality is the proportion of each variable’s variability that is explained by the values: the closer to 1, the better the variable is explained by the factors. Kurtosis is a shape measure that characterizes the flattening of the probability distribution function curve^(8)^.

Communality was also used as an item permanence criterion. Defined as the proportion of common variance present in a given variable, the communality, analyzed together with the factorial load, helps to identify problematic items^(8)^. Although some items presented communality below the cutoff point, factor loadings were adequate and the other tests performed attested to the quality of the model, which supports keeping these items in the instrument.

The factors showed strong internal consistency, according to the CR value. The use of CR instead of Cronbach’s alpha is justified by evidence in the literature that demonstrates its weakness as an indicator of reliability of scores from multidimensional models, due to tau-equivalence:€α considers that all items have equivalent loadings; and suggest new tests, such as CR, a technique that takes into account the factorial load of the items, thus allowing better assessment of the quality of the structural model of psychometric instruments^(20)^.

Different statistical criteria ensured the adequacy of the internal structure estimates, considering their potentials and weaknesses. By using various criteria and indicators, associated with theoretical interpretation, it was possible to eliminate risks and biases and find an adequate factorial solution that was consistent with the original proposal of the instrument. In this sense, we chose to use current and robust techniques, which would only be possible in confirmatory factor analysis: precision, representativeness, sensitivity, replicability, expected factor percentage and model adequacy indexes.

However, some limitations were found, such as the homogeneity of the sociodemographic, economic, and clinical profile of the subjects, who were treated at the same healthcare institution. Although the institution is a reference and treats patients from the North and Northeast regions, the profile was similar among the participants. Such limitation contributed to the exclusion of relevant items for the context of HV, being important to conduct future research involving people with HF of different profiles. Moreover, we should mention the lack of similar instruments in the scientific literature, not allowing for comparisons.

The statistical tests improved the interpretation and reliability of the model and showed that the QVSIC-Human Person showed psychometric parameters of quality and theoretical consistency. The use of the instrument will allow new interpretative horizons on the HV of the person with HF and its results may support care actions by nurses and more effective strategies to reduce the levels of vulnerability.

Additionally, this research brings robust techniques, with methodological rigor and, above all, based on contemporary psychometric recommendations.

## CONCLUSIONS

The QVSIC-Human person, composed of 22 items distributed in 5 dimensions (Water and food intake, Sleep and daily activities, Mental health, Functional health literacy, and Signs and symptoms) showed satisfactory psychometric properties, evidencing a model with good validity and reliability evidences.
